# Outbreak of Middle East Respiratory Syndrome Coronavirus in Camels and Probable Spillover Infection to Humans in Kenya

**DOI:** 10.3390/v14081743

**Published:** 2022-08-09

**Authors:** Isaac Ngere, Elizabeth A. Hunsperger, Suxiang Tong, Julius Oyugi, Walter Jaoko, Jennifer L. Harcourt, Natalie J. Thornburg, Harry Oyas, Mathew Muturi, Eric M. Osoro, John Gachohi, Cynthia Ombok, Jeanette Dawa, Ying Tao, Jing Zhang, Lydia Mwasi, Caroline Ochieng, Athman Mwatondo, Boku Bodha, Daniel Langat, Amy Herman-Roloff, M. Kariuki Njenga, Marc-Alain Widdowson, Peninah M. Munyua

**Affiliations:** 1 Washington State University Global Health Program, Washington State University, Nairobi P.O. Box 72938 00200, Kenya; 2 Paul G. Allen School of Global Health, Washington State University, Pullman, WA 99164, USA; 3 Department of Medical Microbiology and Immunology, University of Nairobi, Nairobi P.O. Box 19676 00100, Kenya; 4 Division of Global Health Protection, U.S. Centers for Disease Control and Prevention-Kenya, Nairobi P.O. Box 40241 00621, Kenya; 5 Division of Viral Diseases, National Center for Immunization and Respiratory Diseases, U.S. Centers for Disease Control and Prevention, Atlanta, GA 30333, USA; 6 Kenya Ministry of Agriculture, Livestock, Fisheries and Cooperatives, Nairobi P.O. Box 30028 00100, Kenya; 7 Dahlem Research School, Freie Universität Berlin, Kaiserswerther Str. 16-18, 14195 Berlin, Germany; 8 School of Public Health, Jomo Kenyatta University of Agriculture and Technology, Nairobi P.O. Box 62000 00200, Kenya; 9 Center for Global Health Research, Kenya Medical Research Institute, Nairobi P.O. Box 54840 00200, Kenya; 10 Kenya Ministry of Health, Nairobi P.O. Box 30016 00100, Kenya; 11 Department of Veterinary and Livestock, County Government of Marsabit, Marsabit 60500, Kenya

**Keywords:** Middle East respiratory syndrome coronavirus, zoonosis, spillover events, MERS-CoV epidemiology, Horn of Africa

## Abstract

The majority of Kenya’s > 3 million camels have antibodies against Middle East respiratory syndrome coronavirus (MERS-CoV), although human infection in Africa is rare. We enrolled 243 camels aged 0–24 months from 33 homesteads in Northern Kenya and followed them between April 2018 to March 2020. We collected and tested camel nasal swabs for MERS-CoV RNA by RT-PCR followed by virus isolation and whole genome sequencing of positive samples. We also documented illnesses (respiratory or other) among the camels. Human camel handlers were also swabbed, screened for respiratory signs, and samples were tested for MERS-CoV by RT-PCR. We recorded 68 illnesses among 58 camels, of which 76.5% (52/68) were respiratory signs and the majority of illnesses (73.5% or 50/68) were recorded in 2019. Overall, 124/4692 (2.6%) camel swabs collected from 83 (34.2%) calves in 15 (45.5%) homesteads between April–September 2019 screened positive, while 22 calves (26.5%) recorded reinfections (second positive swab following ≥ 2 consecutive negative tests). Sequencing revealed a distinct Clade C2 virus that lacked the signature *ORF4b* deletions of other Clade C viruses. Three previously reported human PCR positive cases clustered with the camel infections in time and place, strongly suggesting sporadic transmission to humans during intense camel outbreaks in Northern Kenya.

## 1. Introduction

Middle East respiratory syndrome coronavirus (MERS-CoV) is a betacoronavirus similar to two other recently emergent coronaviruses, severe acute respiratory syndrome coronavirus (SARS-CoV), and SARS-CoV-2, but different because it has a domestic animal reservoir that serves as the primary source of human infections [1,2]. First reported in humans in 2012, MERS-CoV infection causes mild to severe respiratory disease in humans with a case fatality rate of 35%, whereas in camels, the documented infections are mostly asymptomatic with rare cases of rhinorrhea, conjunctivitis, coughing/honking, or diarrhea [3,4,5,6]. As of March 2022, the World Health Organization (WHO) had confirmed more than 2600 human cases and 900 deaths across 27 countries globally, most of them (>80%) in the Middle East and Asia, and none in Africa [7,8]. The global MERS-CoV human case count has been declining since 2016. However, two MERS-CoV cases and one death have been reported in Qatar this year, with both cases reporting contact with camels [9].

Zoonotic transmission of MERS-CoV from camels to humans is often followed by limited human-to-human transmission, occasionally leading to larger chains of transmission in humans and resulting in large clusters of human cases, especially within health care settings [6,10]. Clinical MERS-CoV disease has not been reported in Sub Saharan Africa, where >80% of the world’s dromedary camel population are reared by pastoral communities living in arid regions. Apart from a report of three asymptomatic infections in Kenya and evidence of virus exposure (antibodies) among camel handlers, autochthonous transmission in humans has been rare, despite extensive human–camel contact and >80% MERS-CoV seroprevalence among adult camels [11,12,13,14,15]. This absence of human MERS-CoV disease in Africa may be due to immunological factors, or presence of a genetically different local virus that exhibits high intrinsic adaptation to camels but poor transmissibility or virulence in humans, or both [16,17].

Several groups have conducted community and hospital-based studies in the Horn of Africa (HOA) region, confirming high virus prevalence in camels, but not detecting synchronous infection or clinical disease in humans [11,12,18,19]. Three clades of MERS-CoV circulating in the Middle East, Asia, and Africa have been described, all antigenically similar with capacity to cross-neutralize but with discernable genetic variations in the spike, *ORF3*, and *ORF4b* genes [20,21]. Clade A and B viruses circulate predominantly in the Middle East and Asia, while clade C appears to be found primarily in Africa [20,21,22]. A recent study using ex vivo and in vivo inoculations suggested that the three clades exhibit geographical dominance and that clade C viruses may be less pathogenic than clades A and B [20,21]. To track the circulating variant of MERS-CoV, its transmission dynamics in camels, and investigate zoonotic transmission, we established and followed a prospective cohort of camel calves and their handlers in Northern Kenya.

## 2. Materials and Methods

### 2.1. Study Site, Design, and Enrollment

The study was conducted in Marsabit county in Northern Kenya among communities bordering Ethiopia—an arid region where nomadic pastoral livestock production systems are the main economic activity [23,24]. Inhabitants keep camels, goats, cattle, and sheep, and >80% of the homesteads, often comprising several households, own or manage camels in a herd and share communal resources such as grazing grounds and watering points [25]. The camel population outnumbers humans by 18 to 1 among camel-keeping households [26]. Camel herds are owned or managed by one household or homestead and are usually reared in the ‘home’ herd (consisting of young, pregnant, or nursing camels kept close to the homestead) and the ‘fora’ herd (consisting of the rest of the adult camels herded far away from the homestead) [25].

We identified camel-keeping homesteads within a 50-km radius from Marsabit town through key informant interviews. A homestead was defined as a grouping of dwellings that jointly owned and reared camels in a single herd. Recruitment of camel calves aged 0–24 months was conducted on a rolling basis over the study period. Newborns and calves ≤6 months were preferentially enrolled to increase chances of detecting primary infections before development of immunity from natural infection, as has been shown in other camel studies [27]. Concurrently, we enrolled compound residents who reported any contact with enrolled camels through activities such as milking, herding, feeding, cleaning barns, slaughtering, or grooming camels (this group of people is referred to here as “camel handlers”) within the enrolled homesteads.

### 2.2. Sample Size

To calculate the sample size of the camel cohort, we assumed a MERS-CoV exposure of 77% among infant camels, an annual attack rate of 15%, with a power of 80% at the 95% confidence interval [25,28]. We also accounted for an estimated 40% loss to follow-up during the study. No power calculation was done for the camel handlers and individuals >1 year of age who had contact with enrolled camels [15].

### 2.3. Enrollment, Follow-Up of Camel and Human Cohort and Data Collection

Trained animal health assistants or nurses conducted enrollment and follow-up visits. We used pretested questionnaires on Android^®^ tablets running RedCap^®^ application to collect camel and human data during enrollment and follow-up visits. At enrollment the herd-owner and animal handler were consented, and a baseline questionnaire administered to collect social demographic characteristics of the homestead. We collected data on camel herd structure, herding practices (nomadic or sedentary), and herd mixing while watering or grazing. For each enrolled camel, we captured details such as age, sex, any current illness, if the camel belonged to the ‘home’ herd or the ‘fora’ herd, and we then ear-tagged the calves for ease of follow-up. Camel illness data, collected without knowledge of any infection status, was classified as either a respiratory illness sign (if the camel had nasal discharge, sneezing, cough/honking, or hyper-lacrimation) or other illness sign (if the camel had tick infestation, skin and mouth lesions, lethargy, weight loss, anorexia, or diarrhea). Physical injuries in camels were not documented.

Subsequently, a homestead was visited twice monthly and data on clinical illness in camels was collected in a structured questionnaire, followed by collection of deep nasal swabs from each camel. A follow-up was considered successful when a camel was swabbed, and clinical signs were documented by study staff. Complete follow-up was defined as calves that were available for all their study visits while intermittent follow-up was defined as instances where calves missed some study visits after enrollment but were available for final sample collection at the end of the study period. Loss to follow-up (LTFU) included camels that were not traced for the final sample collection at the end of the follow-up period.

Trained study nurses also collected nasopharyngeal and oropharyngeal swabs from enrolled camel handlers monthly, and when participants called the study toll-free-line to report signs of respiratory illness such as cough, fever, running nose, nasal stuffiness, sore throat, or chest pain. Detailed enrollment and follow-up procedures of camel handlers were as previously described [15].

Data was stored temporarily in RedCap^®^ which was backed up daily in cloud servers. Periodic reports were compiled and shared with the study team and the Ministry of Health.

### 2.4. Sample Collection, Shipping, and Storage

A Dacron swab (Puritan^®^) was used to swab both nostrils of each camel and was immediately placed in cryovials containing viral transport medium. For the camel handlers, nasopharyngeal (NP) and oropharyngeal (OP) swabs were similarly collected using polyester tipped swabs at enrollment and monthly thereafter during the follow-up visits.

All specimen tubes were barcoded and transported to the field laboratory within four hours using cool boxes maintained between 4–8 °C. At the field laboratory, samples were stored temporarily at −160 °C in dry shippers in a field laboratory in Marsabit town for up to 72 h. All specimens were later shipped to the U.S. Centers for Disease Control and Prevention (CDC) supported Kenya Medical Research Institute (KEMRI) laboratories in Nairobi for long-term storage and testing.

### 2.5. Real Time Reverse Transcription PCR Testing

MERS-CoV RNA in camel swabs was detected by real time RT-PCR at the KEMRI-CDC laboratories as described previously [29]. Briefly, total nucleic acids were extracted from 200 µL of the sample, followed by a standard RT-PCR test targeting the envelope (E) and two distinct regions of the nucleocapsid (N) genes. A sample was considered positive if all three PCR targets were positive (defined by a Cycle Threshold/C_T_ value < 40).

### 2.6. MERS-CoV Isolation and Genotypic Analysis

Virus isolation was attempted for all RT-PCR positive camel nasal swabs (C_T_ values below 20) using Vero cells at the biosafety level 3 (BSL-3) KEMRI-CDC laboratory in Kisumu, Kenya. Inoculated cells were incubated at 37 °C for five days and observed daily for cytopathic effects (CPEs). The culture supernatant and cells were examined for the presence of virus by the real time RT-PCR N2 assay targeting the MERS-CoV N gene and immunofluorescence assay using a rabbit antibody against the MERS-CoV N protein [30].

MERS-CoV culture isolates and aliquots of PCR positive samples were selected on the basis of high virus titers (low C_T_ values below 30) for full-genome sequencing, which was performed at the CDC Genomics and Discovery laboratory in Atlanta, USA, following methods described previously [31,32]. Overlapping primer pairs that span the entire MERS-CoV genome were designed and developed against the MERS-CoV reference genome (JX869059). Briefly, RNA was first reverse transcribed by random hexamers to generate MERS-CoV-complementary deoxyribonucleic acid (cDNA) and then amplified using multiple multiplexing PCRs. The resulting multiple PCR amplicon pools from each sample were subject to fragmentation followed by barcoding libraries using the NEBNext Ultra II DNA library prep kit (New England Biolabs, Ipswich, MA, USA) or using the PCR Barcoding Expansion Pack 1–96 (EXP-PBC096) (Oxford Nanopore Technologies, Oxford, United Kingdom). Sequencing was performed using an Illumina MiSeq instrument (San Diego, CA, USA) and the Oxford nanopore GridION. Sanger sequencing was used to fill gaps if initial Illumina sequencing failed to recover the complete genome sequences.

All full sequences were deposited in GenBank (accession numbers OK094446-OK094454). The full and partial genome sequences were aligned in MAFFT v7.013 [33] including sequences from this study and a collection of representative MERS-CoV sequences retrieved from GenBank.

### 2.7. Statistical Analysis

R statistical software version 4.2.1 (R Core Team, Vienna, Austria, 2022) was used to detail the epidemiologic data. Q-GIS version 2.18.15 was used to generate maps. Attack rates were estimated as the proportion of camels with a sample positive for MERS-CoV out of the total number of camels under observation that were tested during the study period. The point estimates and 95% confidence interval for the attack rates estimates were reported. A repeat infection was defined as a camel with a second or third PCR positive sample following at least two successive PCR negative samples. Chi-square test was performed to evaluate for differences among the MERS-CoV infected and re-infected camels compared to non-infected camels, and odds ratios and the corresponding 95% confidence intervals (95% CIs) were reported. Phylogenetic analysis was inferred using the maximum likelihood (ML) method available in PhyML version 3.0 [34], assuming a general time-reversible (GTR) model with a discrete gamma distributed rate variation among sites (Gamma4) and an SPR tree swapping algorithm, and visualized in Mega version 6 [35].

## 3. Results

### 3.1. Description of Homesteads, Camel Herds, and the Enrolled Calves

From April 2018 to March 2020, we enrolled and followed 243 camel calves (18.5%) from a total of 1311 camels in 33 homesteads. About half of the camel calves (51.0%) were females, and the median age of the cohort at enrollment was four months (interquartile range, IQR = 1–9 months). Most of the enrolled camels (88.1%) were maintained close to the homestead in the home herd.

**Figure 1:**
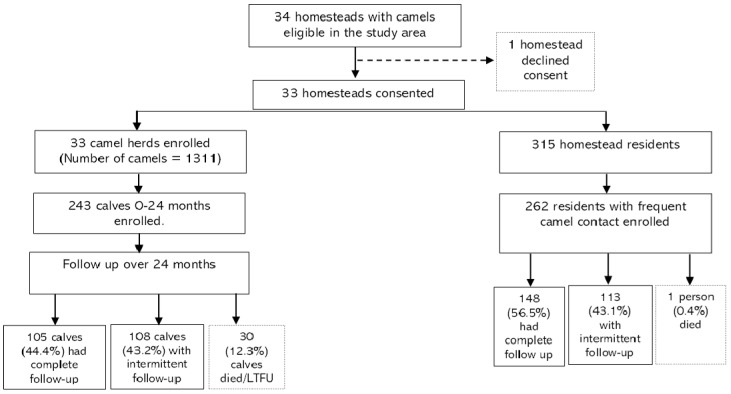
Study flow chart showing screening, enrollment, and follow-up of the linked camel–human cohort.

Almost all camel herds (>90%) practiced communal grazing with several other herds and shared watering points. Seven of the 33 homesteads (21.9%) acquired camels through purchase into their herds during the two-year study period, although the new camels were not enrolled in the cohort. The number of follow-up visits after enrollment ranged between 0–45 visits per camel, with a median of 17 follow-up visits (IQR = 7–31). In total, 105 calves (44.4%) had a complete follow-up, 108 calves (43.2%) were available intermittently over the study period, 30 calves died, and 16 were lost to follow-up immediately after enrollment (Figure 1). Overall, the cohort was followed up for a total of 270.6 camel-years equivalent to a median of 16 follow-up visits per camel (interquartile range, IQR = 6–30). A total of 30 calves died during the two-year follow-up, almost all (90.0%) due to predators and injuries.

### 3.2. Clinical Disease in Camels

**Figure 2:**
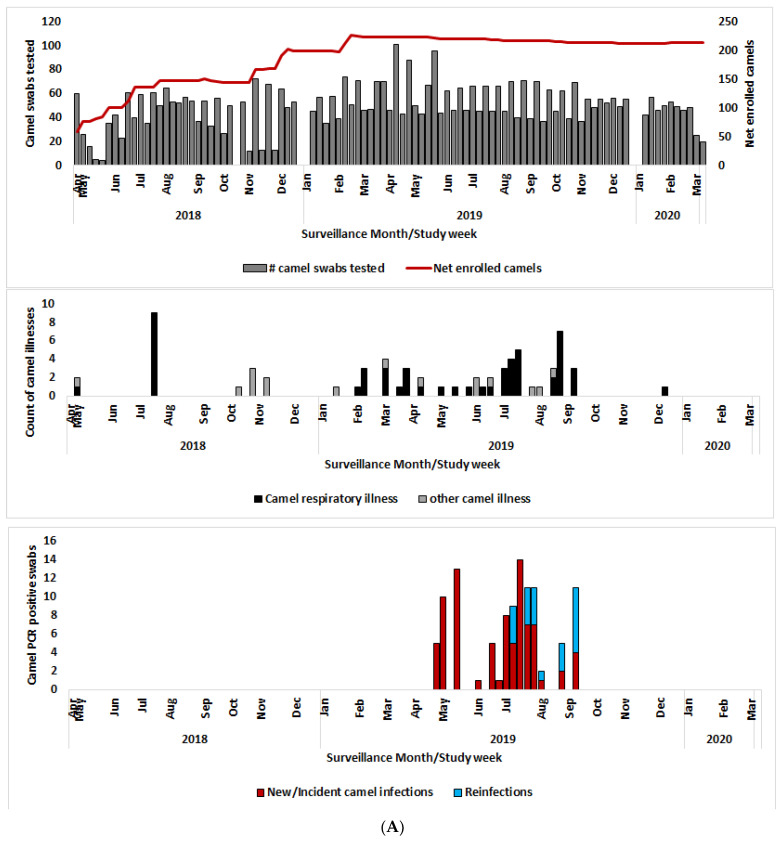

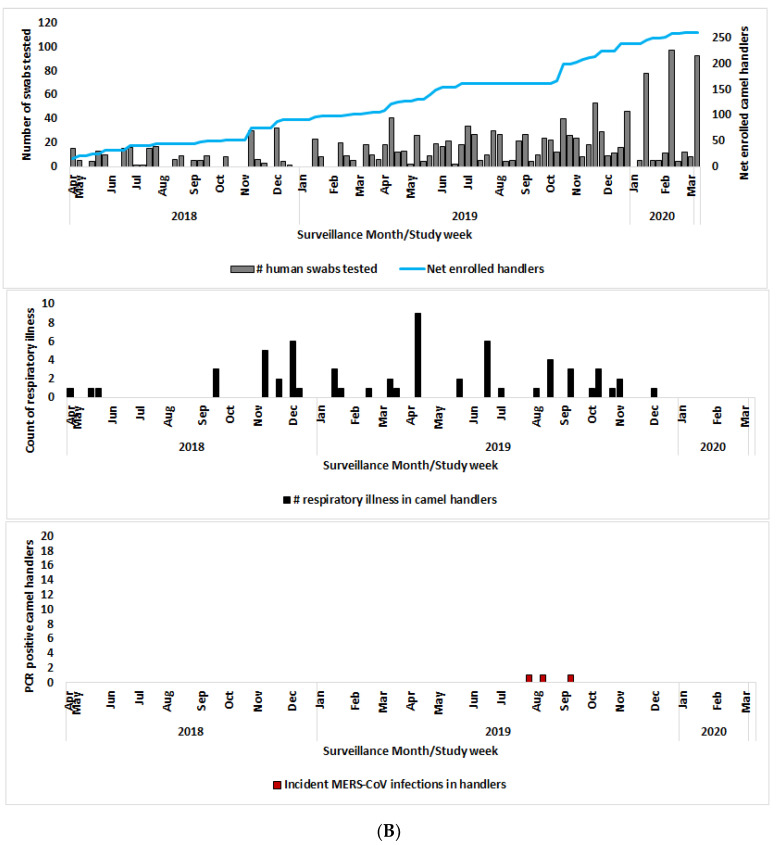
(**A**) Net enrollment, number of swabs tested, respiratory illness signs, and PCR positive swabs enrolled camels; (**B**) Net enrollment, number of swabs tested, respiratory illness signs, and PCR positive swabs enrolled camel handlers.

During the 2-year follow-up period, 68 illness episodes were recorded in 58 (23.9%) of the 243 camels, 70% of them between April and September 2019 (Figure 2A). A total of 52 illness episodes (76.5%) were respiratory illness signs, comprised of increased nasal discharge (28/52 or 53.8%), hyper-lacrimation (48.1%), coughing/honking (15.4%), and sneezing (3.8%). The respiratory illness incidence rate was 192.2 episodes per 1000 camel years. The remaining 16 non-respiratory illnesses comprised of anorexia (8/68 or 11.8%), lethargy (8.8%), enlarged lymph nodes (8.8%), weight loss (8.8%), diarrhea (7.4%), and skin and oral lesions (5.9%).

### 3.3. Confirmation of MERS-CoV Outbreak in Camels

Of the 4692 nasal swabs collected and tested between April 2018 and March 2020, 124 swabs (2.6%) collected from 83 calves in 15 homesteads were positive for the MERS-CoV virus by RT-PCR. All the positive samples were collected between April and September 2019, the same period when >70% of the camel clinical disease was documented, henceforth referred to as the outbreak period (Figure 2A). During the outbreak period, 162 calves (66.7% of enrolled cohort) from 20 homesteads (60.6% of enrolled homesteads) were sampled and a total of 1853 swabs (39.5% of total swabs) were collected, or a median of 14 samples per camel (IQR 5–16). During this outbreak period, completeness of sampling did not vary by age or sex (Table 1).

The 83 calves involved in the outbreak were drawn from 15 of the 20 homesteads on follow-up at that time, giving a camel attack rate of 51.2% (95% CI 43.3–59.2%) and herd attack rate of 75.0% (95% CI 50.9–91.3%). The duration of viral RNA detection in a camel during the outbreak ranged from 2–6 weeks (Supplementary Figure S1). PCR detection in camels did not vary by sex, however, camels aged between 5–12 months were more likely to be infected when compared to camels from birth–4 months (odds ratio, OR = 3.21, 95% CI = 1.08–9.56 and *p* = 0.04).

Of the 83 PCR-positive calves, 20 (24.1%) had clinical signs of illness within 2 weeks of the sampling date, and of these, 19 calves had respiratory signs, including nasal discharge, coughing, and increased lacrimation.

### 3.4. Reinfections

Of the 83 PCR positive calves, 61 (73.5%) were infected once, 22 (26.5%) were reinfected, while one camel had three separate infections, giving a total of 106 separate infections (Table 2 and Supplementary Table S2). The median duration between re-infections was 59 days (IQR 49.5–78.5). The median age of the calves at first infection was 16 months (IQR = 7–22 months). The distribution of reinfected camels is shown in Table 2. Compared to females, male calves were more likely to be reinfected (OR = 2.88, 95% CI 1.03–8.09) and the difference was statistically significant. By age, calves 13–18 months were more likely to be re-infected compared to calves 0–4 months (OR = 5.83, 95% CI 1.15–37.93).

### 3.5. Human MERS-CoV Infections and the Spatial and Temporal Correlation with the Camel Outbreak

We previously reported three asymptomatic human MERS-CoV infections by RT-PCR from the cohort of 262 camel handlers [15]. The rest of the humans, sampled monthly, were negative for MERS-CoV by PCR (Figure 2B). Here, we build on this by correlating the spatial and temporal occurrence of human clinical illness within the handler’s cohort (*n* = 262) with the MERS-CoV outbreak in their camels.

**Figure 3:**
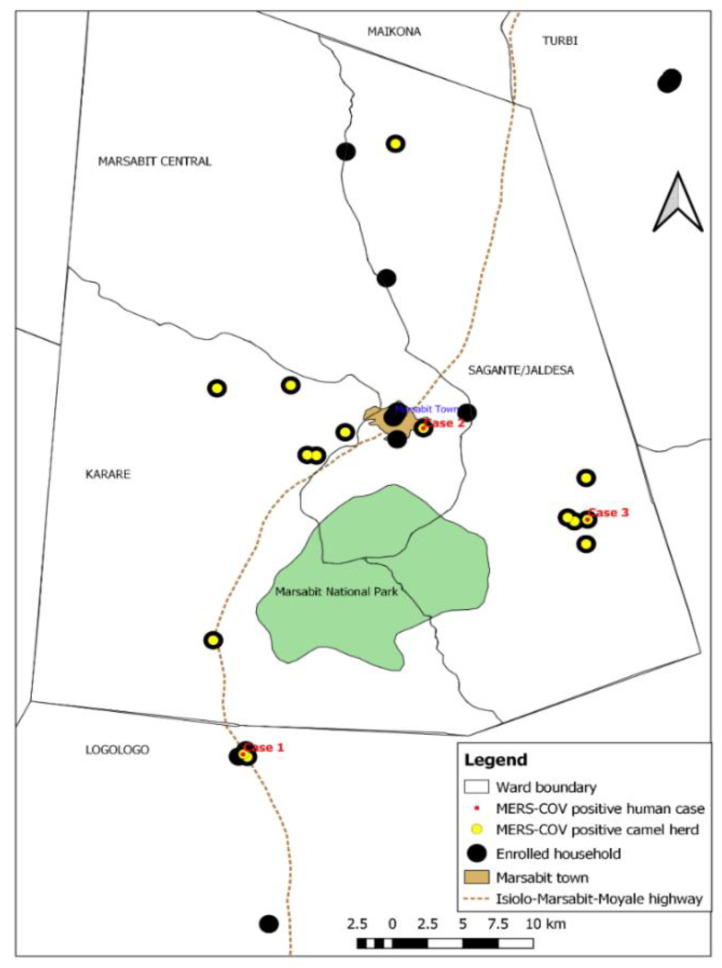
Spot-map of the study area showing the location of enrolled camel homesteads (black spots), herds involved in the MERS-CoV outbreak (yellow spots), and human infections (red dots). The three human MERS-CoV infections clustered with camel infections.

When analyzed alongside the camel data, the first human case (Case 1) tested positive in the last week of July 2019 and was a 20-year-old female spouse of a camel handler with two camels, one of which was enrolled in the cohort study, but this camel did not test PCR positive. However, two neighboring compounds (<2.5 Km away) with 14 enrolled calves had positive camels, while Case 1 tested positive. Case 2 was a 50-year-old male camel herder, diagnosed PCR positive in mid-August 2019, two weeks after one of the enrolled camels in his homestead was also tested positive. Case 3 was a 24-year-old camel herder who tested positive on 12th September 2019, the same day when three out of four camels sampled in his homestead also tested positive (Figure 3, Supplementary Table S3 and Supplementary Figure S1).

### 3.6. Whole Genome Sequencing of MERS-CoV Isolates from Camels

**Figure 4:**
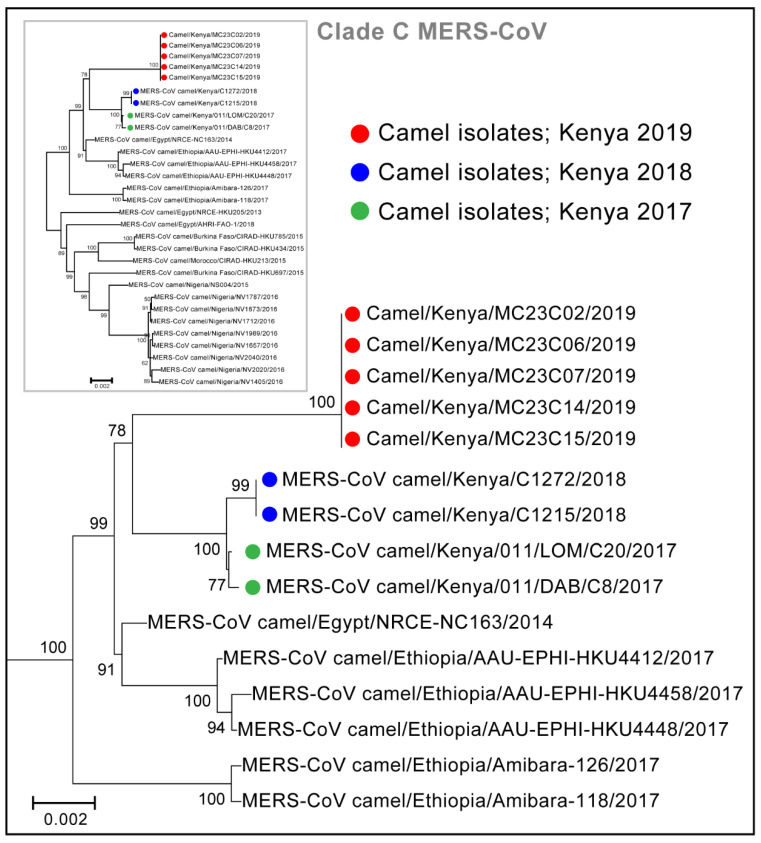
Phylogenetic tree of camel-derived MERS-CoV sequences from Clade C. The sequences indicated by the green and blue colored dots were isolated from camels in Kenya in earlier studies. Sequences denoted by the red dots were isolated from our study and were based on whole genome sequences (sequences measuring 30,112 kilobases).

From six nasal swab samples, taken from six different camels, that had C_T_ values <20, we obtained four virus isolates. The virus isolates and eight additional PCR positive swabs from seven camels (Ct < 30) were subjected to whole genome sequencing. A total of nine whole-genome sequences were generated (sequence measuring 30,112 kilobases) representing sequences from five unique camels in a herd that was involved in the May 2019 outbreak peak. Four camels had two sequences generated from isolates derived from nasal swabs collected at two successive sampling points. One camel had a sequence determined directly from a nasal swab. All nine genome sequences in this study were identical and were clustered with the clade C, alongside other African MERS-CoV (Figure 4 and Supplementary Figure S4). Within Clade C, the new Kenyan strains clustered with sub-clade C2 alongside other strains from Kenya and Ethiopia collected in 2017 and 2018. All the nine genomes from this study had identical 12-nucleotide deletions near the 3′ end of OFR3, resulting in nucleic acid deletion between 25,792–25,803 and causing 4 amino acid deletion (*ORF3: 87T-*, *88E-*, *89H-*, *90V-*) compared to the GenBank sequence JX869059). The 12 nucleotide deletions started slightly (6 nucleotides) upstream of a 42-nucleotide deletion identified in Clade C1.1 strains from camels in Nigeria in 2016 (Supplementary Figure S2). These *ORF3* deletions did not affect the stop codon and were not detected in Clade A and B viruses. We did not detect previously reported deletions in *ORF4b* of Clade C viruses. Our strains also had one nucleotide insertion at the 5′ non-coding region, which was also detected in viruses collected in Ethiopia in 2017 but not in the older Kenya viruses nor in other Clade C viruses.

## 4. Discussion

In this two-year cohort study, we detected an outbreak of MERS-CoV infection among 243 camels in Northern Kenya, affecting up to three-quarters of homesteads under surveillance. This camel outbreak was accompanied by increased respiratory illness signs and possibly repeat infections in camels, and likely resulted in acute camel-to-human MERS-CoV transmission events in three camel handlers as previously reported [15]. Sequencing of the camel isolates revealed genetically distinct clade C sub clade C2 MERS-CoV virus closely related to other sequences from the region. Spatial and temporal clustering of camel and human MERS-CoV cases strongly supports sporadic zoonotic spillover of Clade C MERS-CoV virus, as suggested in other studies [14,15,36].

Despite intense camel infection, and regular swabbing of handlers, only three positive but asymptomatic camel handlers were detected, suggesting that camel-to-human transmission is uncommon and not severe in this setting. This result is in agreement with a number of studies in the Horn of Africa region showing high anti MERS-CoV seroprevalence in camels but minimal detection of the virus or antibodies in humans [15,25]. A study of camels, other livestock, and wildlife in Sudan and Qatar found widespread camel exposure but no human infection [19]. Another study conducted in 2013 by our group found 18 out of 760 (2.4%) people positive for MERS-CoV antibodies by ELISA but negative by neutralization test, suggesting low level immune responses consistent with the possibility of asymptomatic infections [12]. In a past study, the three camel handlers were reported to be either young or middle-aged and were all asymptomatic [15]. We have not been able to explore risk factors for human infection due to the small number of infected camel handlers who all reported frequent camel contact.

The camel MERS-CoV outbreak was accompanied by increased respiratory illness signs in camels across the three outbreak peaks, signaling potential increased shedding events from camels that could contribute to transmission to camels and humans. A few studies have shown that camels infected with MERS-CoV may show a mild clinical illness. In this study, we were able to pick an increased intensity of reporting of camel illness, mostly mild respiratory signs which may underscore a possible role of syndromic surveillance in camels in this region [27,37,38,39]. The onset of the outbreak in April coincided with the long rains and peak calving season in northern Kenya. This season is usually characterized by frequent herd mixing during the *Sorio* traditional ceremony, celebrated in March-April when all livestock are brought back to the homestead for communal celebrations, creating a possible trigger of disease outbreaks [25]. However, even without cultural events such as *Sorio*, camel herds are reared in a nomadic pastoralist system with frequent mixing at watering points and grazing fields, providing ample opportunities for inter-herd transmission.

Within our camel cohort, the attack rate was high across all age groups in this study. However, the attack rate was highest among the 5–12-month-old camels, possibly reflecting a first infection in a young susceptible cohort following the waning of maternally derived antibodies. At 6–12 months, camel calves reared in the range production system are weaned and leave the *home* heard to join adult camels in the *fora* herd, possibly exposing them to infections once protection from the maternally derived antibodies wanes [25]. Past studies in the Middle East have shown widespread infections of young camels during outbreaks and suggested the possibility of reinfections [27,38,40].

We also observed a likely < 2-week to 6-week period of virus shedding for most camels, although repeat infections were common (a quarter of infected camels had repeat infections). The fact that even second or third MERS-CoV detections were clustered in time suggests that these were more likely repeat infections than prolonged or intermittently shedding. Additionally, CT values for the positive results also showed high viral shedding (heavy viral load), which is consistent with primary shedding as opposed to contamination. Lately, in the case of SARS-CoV-2, it has become apparent that coronaviruses can indeed cause multiple repeat infections [41]. The repeat infections and high attack rates could explain the protracted outbreak that lasted 5 months and could underlie the mechanism of virus maintenance in camels.

Our study found the clade C sub-clade C2 MERS-CoV to be responsible for this outbreak. Few recent studies have associated the African MERS-CoV strains with deletions in accessory genes, particularly *ORF3* and *ORF4b* genes, that are likely to affect viral replication and pathogenicity [18,20,21,22]. Clade C1 viruses from West and North Africa, which have deletions in *ORF4b*, exhibited lower replication in human cells and in ex vivo human bronchus and lung tissues [20]. This study and others show deletions near the 3′ end of *ORF3*, however, the importance of this on virus replication and pathogenicity has not been determined [21,22]. The likely explanations behind presence of only clade C viruses in the Horn of Africa remain elusive. First, although MERS-CoV viruses may have originated from the Horn of Africa, with serologic evidence of the virus in camels detected >30 years before confirmation of the first human clinical cases of MERS-CoV [42] studies including this one have not detected the entire spectrum of the viruses in the region [13,21]. Second, clade C African viruses have not been detected in the Middle East, despite the fact that >60% of camels consumed in that region are imported from the Horn of Africa [22].

This study had some limitations. The biweekly follow-up schedule limited our ability to accurately characterize the natural history of MERS-CoV infection in camels. This was further compounded by the intermittent follow-up of about half of the enrolled camels. Being a nomadic pastoralist community, animals and people move over large distances for water and livestock feeding grounds, and this contributed to significant loss to follow-up, especially among adult camels that are also more difficult to restrain and sample. It is possible that some infections were missed between the biweekly sampling dates, especially if viral shedding was for a short period, as shown in this and other past studies [27,39]. The lack of serological data for camels and humans in this paper limited our ability to verify missed infections by checking for seroconversion, and therefore our attack rates may be underestimated. As we did not enroll all camels in herds, some infections would also have been missed in adult camels, even if the calves in our cohort were not infected.

## 5. Conclusions

In conclusion, our study describes the infection and transmission dynamics of MERS-CoV among camels and closely associated humans in the Horn of Africa. These findings shed light on the natural maintenance and transmission cycle of the virus in the region, perhaps driven by pathogen fidelity, geography, and host factors. Our study showed high levels of clade C MERS-CoV virus transmission among East African camels, with sporadic spillover to persons with camel contact. The possibility of virus evolution in the face of high transmission and the possible introduction of other clades through camel and human movements across the Arabian Peninsula underscores the need for continued virologic and serologic surveillance in camels and humans to understand the public health implications of MERS-CoV at the human–camel interface in Africa [1,21,43].

## Figures and Tables

**Table 1:** Attack rates by age and sex among enrolled calves tested at least once during the MERS-CoV outbreak period in Northern Kenya, 2019 (*n* = 162).

Variable	Camels on Follow-Up during Outbreak Period ^ϕ^ (*n* = 162)	Swabs Collected (April–September 2019) (*n* = 1853)	Median Swabs per Calf (IQR)	PCR Positive (1st PCR Positive) *n* = 83	Attack Rates ^¶^ % (95% CI)	*p*-Value
By Sex						
Male	86 (53.1%)	963 (52.0%)	14 (5–16)	41 (49.4%)	47.7% (36.8, 58.7)	Ref
Female	76 (46.9%)	890 (48.0%)	14 (5–16)	42 (50.6%)	55.3% (43.4, 66.7)	0.93
By Age (in months) ^ƛ^						
0–4	57 (35.2%)	595 (32.1%)	15 (5–16)	24 (28.9%)	42.1% (29.1, 55.9)	Ref
5–12	20 (12.3%)	285 (15.4%)	16 (15–16)	14 (16.9%)	70.0% (45.7, 88.1)	0.04 ^µ^
13–18	45 (27.8%)	481 (26.0%)	13 (5–15)	22 (26.5%)	48.9% (33.7, 64.2)	0.49
19+	40 (24.7%)	492 (26.6%)	14 (11–15)	23 (27.7%)	57.5% (40.9, 72.9)	0.14
All calves	162 (100.0%)	1853 (100.0%)	14 (5–16)	83(100.0%)	51.2% (43.3, 59.2)	NA

^ϕ^ 162 calves included in the analysis were sampled during the outbreak period (April–September 2019); ^¶^ Attack rates were computed for the first infections; ^ƛ^ Calf age as at end of March 2019; ^µ^ Fisher’s exact test (FET).

**Table 2:** Repeat infections among camel calves during an outbreak of MERS-CoV in camels in Northern Kenya, *n* = 22.

Variable	Camels with Repeat Infections * *n* (%)	OR (95% CI)	*p*-Value
Sex			
Female	7 (31.8%)	Ref	
Male	15 (68.2%)	2.88 (1.03–8.09)	0.04
By Age (months)			
0–4	3 (13.6%)	Ref	
5–12	1 (4.5%)	0.54 (0.01–7.67)	1.00
13–18	10 (45.5%)	5.83 (1.15–37.93)	0.02
19+	8 (36.4%)	3.73 (0.72–24.80)	0.09
All calves	22 (100.0%)		

* Repeat infection was defined as a camel with a second or third PCR positive sample following at least two successive negative samples.

## Data Availability

All data underlying this article are available in the article.

## Supplementary Materials

The following supporting information can be downloaded at: https://www.mdpi.com/article/10.3390/v14081743/s1, Figure S1: Sequence index plots of MERS-CoV infection in camels and camel handlers in 15 homesteads; Figure S2: The genetic sequences of the Kenya MERS-CoV virus sequences (bottom of the list) showing 12 nucleotide deletions at the 3′ region of the *ORF3* (arrow). Available sequences from African camels and the Middle East have also been included for comparison purposes; Table S1: Distribution of PCR Positivity by enrolled herd among 33 camel herds in Marsabit; Table S2: Dates of PCR positive results and cycle threshold values for calves involved in multiple outbreak peaks, *n* = 22. Reinfections are shown in brown cell shade; Table S3: Demographic characteristics, clinical and contact history of the 3 human MERS-CoV cases detected in Northern, Kenya. This updated table is adopted from Munyua et al., 2021 [15]; Table S4: Comparison of MERS-CoV genetic sequences from nasal swabs and culture isolates of camel samples from Marsabit, Kenya.
