# Supplementary material for: Outbreak of Middle East Respiratory Syndrome Coronavirus in Camels and Probable Spillover Infection to Humans in Kenya
Source: Viruses. 2022 Aug 9;14(8):1743. doi: 10.3390/v14081743 (PMC9413448; doi:10.3390/v14081743)
Supplement: Supplementary file 1 [file viruses-14-01743-s001.zip › viruses-1852885-supplementary/Supplementary materials/Supplementary tables.pdf]

Supplementary Tables

Supplementary Table S1: Distribution of PCR Positivity by enrolled herd among 33 camel herds in Marsabit

Herd Number	Estimated herd size (a)	Enrolled calves (b)(%)	No. of PCR Positive Calves (c)	Percent positive (c/b)%
M01	19	17	1	5.9%
M02	20	18	8	44.4%
M03	15	8	5	62.5%
M04	31	21	9	42.9%
M05	40	31	18	58.1%
M06	46	15	0	0.0%
M07	32	6	3	50.0%
M08	14	3	0	0.0%
M09	32	3	0	0.0%
M10	89	6	0	0.0%
M11	39	2	0	0.0%
M12	101	3	0	0.0%
M13	40	7	0	0.0%
M14	21	6	5	83.3%
M15	21	2	0	0.0%
M16	20	1	0	0.0%
M17	10	1	0	0.0%
M18	60	1	0	0.0%
M19	22	10	6	60.0%
M20	24	4	2	50.0%
M21	37	5	2	40.0%
M22	75	1	0	0.0%
M23	50	16	12	75.0%
M24	39	5	0	0.0%
M25	40	9	4	44.4%
M26	14	2	0	0.0%
M27	25	5	3	60.0%
M28	22	2	2	100.0%
M29	44	3	0	0.0%
M30	97	1	0	0.0%
M31	50	16	0	0.0%
M32	120	12	3	25.0%
M33	2	1	0	0.0%
**Total for 33 herds**	**1311**	**243**	**83**	**34.2%**

Supplementary Table S2: Dates of PCR positive results and cycle threshold values for calves involved in multiple outbreak peaks, n=22. Reinfections are shown in brown cell shade

Camel ID	1st PCR Positive result				2nd PCR Positive result				3rd PCR Positive Result				4th PCR Positive Result			
	Follow up No./date (2019)		CT Values		Follow up No./date (2019)		CT Values		Follow up No./date (2019)		CT Values		Follow up No./date (2019)		CT Values	
	FU No.	Date	NCV_E	NCV-N2	FU No.	Date	NCV_E	NCV-N2	FU No.	Date	NCV_E	NCV-N2	FU No.	Date	NCV_E	NCV-N2
M02C15	8	3rd Jun	20.62	18.68	12	1st Aug	29.96	27.47	NA	NA	NA	NA	NA	NA	NA	NA
M03C02	27	14th May	22.47	20.56	33	1st Aug	36.39	33.53	NA	NA	NA	NA	NA	NA	NA	NA
M03C03	33	1st Aug	38.34	38.52	36	12th Sept	36.1	32.81	NA	NA	NA	NA	NA	NA	NA	NA
M03C07	27	14th May	36	34.63	35	29th Aug	21.74	20.92	NA	NA	NA	NA	NA	NA	NA	NA
M03C08	26	25th Apr	29.9	22.55	33	1st Aug	36.55	36.27	36	12th Sept	34.49	32.13	NA	NA	NA	NA
M04C20	11	16th Jul	18.01	16	15	10th Sept	32.34	30.72	NA	NA	NA	NA	NA	NA	NA	NA
M05C30	6	13th May	17.07	16.46	11	22nd Jul	22.9	23.29	NA	NA	NA	NA	NA	NA	NA	NA
M07C05	33	31st Jul	35.86	32.39	36	12th Sept	35.74	33.01	NA	NA	NA	NA	NA	NA	NA	NA
M14C03	28	17th Jun	32.06	35.15	33	26th Aug	34.29	34.12	NA	NA	NA	NA	NA	NA	NA	NA
M14C05	25	13th May	17.99	15.12	33	26th Aug	30.66	29.3	NA	NA	NA	NA	NA	NA	NA	NA
M21C04	29	30th Jul	21.68	19.54	32	11th Sept	16.92	14.26	NA	NA	NA	NA	NA	NA	NA	NA
M23C04	21	30th Apr	38.25	36.74	22	14th May	40.1	36.05	26	10th Jul	27.53	26.18	NA	NA	NA	NA
M23C05	21	30th Apr	37.2	35.83	22	14th May	33.24	33.06	26	10th Jul	37.87	36.78	NA	NA	NA	NA
M23C06	21	30th Apr	21.87	20.22	22	14th May	38.6	38.7	26	10th Jul	20.27	17.53	NA	NA	NA	NA
M23C07	21	30th Apr	15.08	14.5	23	28th May	36.62	37.95	26	10th Jul	34.9	33.07	NA	NA	NA	NA
M23C08	21	30th Apr	35.94	34.24	27	23rd Jul	37.85	32.24	NA	NA	NA	NA	NA	NA	NA	NA
M23C14	5	30th Apr	19.18	16.94	7	28th May	35.05	35.05	11	23rd Jul	25.52	25.49	NA	NA	NA	NA
M23C15	5	30th Apr	18.83	20.54	11	23rd Jul	28.1	20.86	NA	NA	NA	NA	NA	NA	NA	NA
M25C05	21	14th May	22.78	18.67	27	31st Jul	33.94	31.19	NA	NA	NA	NA	NA	NA	NA	NA
M25C08	21	14th May	16.41	15.41	27	30th Jul	19.62	15.72	NA	NA	NA	NA	NA	NA	NA	NA
M28C01	16	4th Jul	36.09	32.55	17	17th Jul	37.83	35.85	18	31st Jul	33.74	29.21	21	11th Sept	30.55	28.64
M28C02	15	20th Jun	19.82	17.58	16	4th Jul	38.87	39	21	11th Sept	30.84	30.94	NA	NA	NA	NA

Supplementary Table S3: Demographic characteristics, clinical and contact history of the 3 human MERS-CoV cases detected in Northern, Kenya. (Updated table adopted from Munyua et al, 2021)[15]

**Variable**	**Case 1**	**Case 2**	**Case 3**
Age (yrs.)	20	50	24
Sex	Female	Male	Male
Herd Name	Kamboe (M33)	Quachacha (M21)	Kubi Onre(M07)
Occupation	Spouse to a camel herder	Camel herder	Camel herder
Enrolled on (Date)	6/23/2019	5/21/2019	5/2/2018
PCR Positive on (Date)	7/31/2019	8/13/2019	9/12/2019
Number of follow ups	3	3	16
Clinical signs on day of sampling	None	None	None
Underlying illness (e.g., HIV, TB, Cancer), Smoking or Travel history	None	None	None
Number of household occupants	12	10	9
Any ill HH member?	No	No	No
Type of camel contact	Infrequent camel contact	Herding, Milking, Feeding, Grooming, Cleaning barns	Herding, Milking, Feeding, Grooming, Cleaning barns
Contact with PCR Positive camels in herd/village	1 PCR positive camel in a herd <2.5Km away	1 PCR positive camel in the herd 2 weeks prior	3 PCR positive camels in the herd on same day

Supplementary Table S4: Comparison of MERS-CoV genetic sequences from nasal swabs and culture isolates of camel samples from Marsabit, Kenya

Camel ID	Date of camel sampling	Sample type	Sequence	Sequence comparison for full genome and S gene
M23C06	4/30/2019	Nasal Swab	full genome	identical except mixed bases (2481Y)
M23C06	4/30/2019	isolate	full genome	identical except mixed bases (2481T)
M23C06	5/14/2019	Nasal Swab	not available	
M23C04	4/30/2019	Nasal Swab	partial S gene	identical
M23C04	5/14/2019	Nasal Swab	partial S gene	
M23C05	5/14/2019	Nasal Swab	partial S gene	1018/22473R[1]
M23C05	4/30/2019	Nasal Swab	partial S gene	1018/22473G[1]
M23C07	4/30/2019	Nasal Swab	full genome	identical except mixed bases (2481T, 11786C)
M23C07	4/30/2019	isolate	full genome	identical except mixed bases (2481Y, 11786Y)
M23C07	5/28/2019	Nasal Swab	partial S gene	identical based on available sequences
M23C09	4/30/2019	Nasal Swab	partial S gene	2212/23667C[1]
M23C09	5/14/2019	Nasal Swab	partial S gene	2212/23667Y[1]
M23C15	4/30/2019	Nasal Swab	full genome	identical except mixed bases (2481Y)
M23C15	4/30/2019	isolate	full genome	identical except mixed bases (2481T)
M23C14	4/30/2019	Nasal Swab	full genome	identical except mixed bases (2481T, 26219C,26294R)
M23C14	4/30/2019	isolate	full genome	identical except mixed bases (2481Y, 26219Y,26294A)
M23C14	5/28/2019	Nasal Swab	partial S gene	identical based on available sequences
M23C01	4/30/2019	Nasal Swab	partial S gene	identical based on available sequences
M23C02	4/30/2019	Nasal Swab	full genome	identical based on available sequences
M23C08	4/30/2019	Nasal Swab	partial S gene	identical based on available sequences

[1]position of S/genome
