# Supplementary figures and images for: Outbreak of Middle East Respiratory Syndrome Coronavirus in Camels and Probable Spillover Infection to Humans in Kenya
Source: Viruses. 2022 Aug 9;14(8):1743. doi: 10.3390/v14081743 (PMC9413448; doi:10.3390/v14081743)

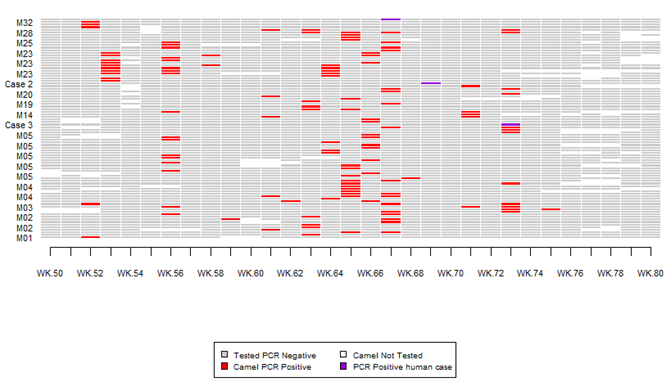

Supplement: Supplementary file 1 [file viruses-14-01743-s001.zip › viruses-1852885-supplementary/Supplementary materials/Supplementary Figure S1.tif]

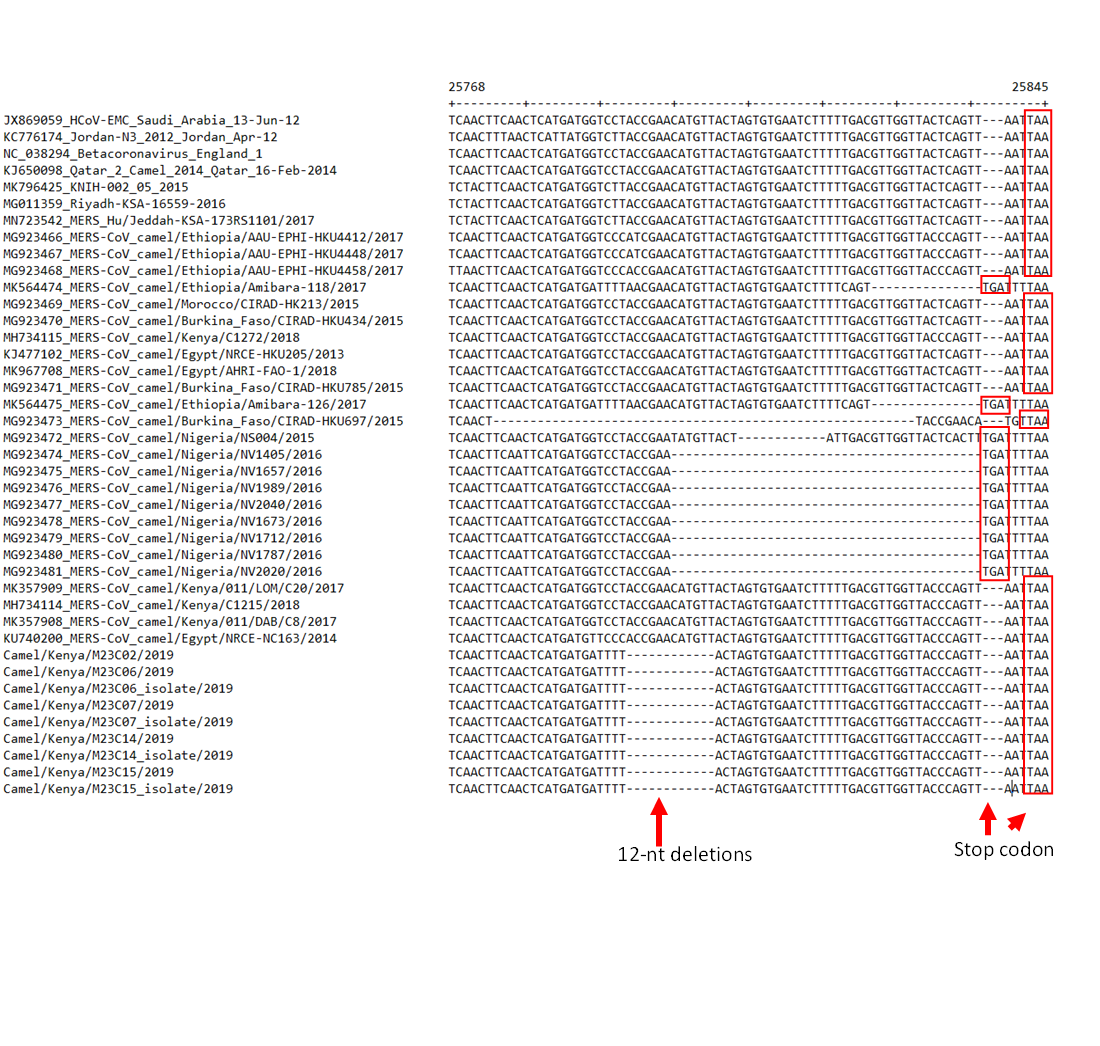

Supplement: Supplementary file 1 [file viruses-14-01743-s001.zip › viruses-1852885-supplementary/Supplementary materials/Supplementary Figure S2.tif]
